# Reducing Ageism: PEACE (Positive Education about Aging and Contact Experiences) Model

**DOI:** 10.1093/geroni/igaa057.2226

**Published:** 2020-12-16

**Authors:** Sheri Levy, Ashley Lytle, Jamie Macdonald, MaryBeth Apriceno

**Affiliations:** 1 Stony Brook University, Stony Brook, New York, United States; 2 Stevens Institute of Technology, Hoboken, New Jersey, United States

## Abstract

Drawing on interdisciplinary theorizing and research, the PEACE (Positive Education about Aging and Contact Experiences) model points to two interrelated factors that reduce ageism: providing education about aging and positive intergenerational contact experiences with older adults (Levy 2016). Evidence supporting the model will be discussed including a semester-long pre-posttest intervention with undergraduates (non-gerontology course) who learned about aging and had face-to-face and Instagram contact with older adults (Lytle, Nowacek, & Levy, 2020), brief online pre-post test experimental-control group studies with undergraduates and a community sample who learned about aging and positive intergenerational contact experiences (Lytle & Levy, 2017), and an online experimental-control group study with undergraduates who viewed brief videos addressing PEACE model components (Lytle, Macdonald, Apriceno, & Levy, under review). Across studies, PEACE model interventions promoted increased aging knowledge as well as reduced negative stereotyping of older adults, aging anxiety, and concerns about aging. Future directions will be discussed.

